# Crosstalk between efferocytic myeloid cells and T-cells and its relevance to atherosclerosis

**DOI:** 10.3389/fimmu.2024.1403150

**Published:** 2024-05-30

**Authors:** David Ngai, Santosh R. Sukka, Ira Tabas

**Affiliations:** ^1^ Department of Medicine, Columbia University Irving Medical Center, New York, NY, United States; ^2^ Department of Pathology and Cell Biology, Columbia University Irving Medical Center, New York, NY, United States; ^3^ Department of Physiology, Columbia University Irving Medical Center, New York, NY, United States

**Keywords:** efferocytosis, myeloid, T-lymphocytes, crosstalk, inflammation, atherosclerosis, resolution

## Abstract

The interplay between myeloid cells and T-lymphocytes is critical to the regulation of host defense and inflammation resolution. Dysregulation of this interaction can contribute to the development of chronic inflammatory diseases. Important among these diseases is atherosclerosis, which refers to focal lesions in the arterial intima driven by elevated apolipoprotein B-containing lipoproteins, notably low-density lipoprotein (LDL), and characterized by the formation of a plaque composed of inflammatory immune cells, a collection of dead cells and lipids called the necrotic core, and a fibrous cap. As the disease progresses, the necrotic core expands, and the fibrous cap becomes thin, which increases the risk of plaque rupture or erosion. Plaque rupture leads to a rapid thrombotic response that can give rise to heart attack, stroke, or sudden death. With marked lowering of circulating LDL, however, plaques become more stable and cardiac risk is lowered—a process known as atherosclerosis regression. A critical aspect of both atherosclerosis progression and regression is the crosstalk between innate (myeloid cells) and adaptive (T-lymphocytes) immune cells. Myeloid cells are specialized at clearing apoptotic cells by a process called efferocytosis, which is necessary for inflammation resolution. In advanced disease, efferocytosis is impaired, leading to secondary necrosis of apoptotic cells, inflammation, and, most importantly, defective tissue resolution. In regression, efferocytosis is reawakened aiding in inflammation resolution and plaque stabilization. Here, we will explore how efferocytosing myeloid cells could affect T-cell function and vice versa through antigen presentation, secreted factors, and cell-cell contacts and how this cellular crosstalk may contribute to the progression or regression of atherosclerosis.

## 1 Introduction

Appropriate regulation of inflammation and its resolution is critical for host defense and maintaining tissue homeostasis. Communication between the innate arm of immunity, particularly myeloid cells, notably macrophages and dendritic cells, and the adaptive arm of immunity, notably T-lymphocytes, is essential. Dysregulation of myeloid-T-lymphocyte crosstalk can contribute to the pathogenesis and exacerbation of chronic inflammatory diseases such as atherosclerosis (1). Atherosclerosis is characterized by the formation of a fibro-fatty plaque in the arterial wall consisting of a necrotic core made up of deposited lipids and dead cells, immune cells including myeloid cells and T-lymphocytes, and a fibrous cap overlaying the necrotic core (2). Atherosclerosis progression is driven by elevated circulating LDL levels that cause increased lipid deposition and inflammatory cell infiltration into the plaque (2). Atherosclerosis regression on the other hand is a clinically relevant phenomenon that occurs with marked lowering of circulating LDL, which promotes inflammation resolution, reduced necrotic core size, and thickening/strengthening of the fibrous cap (3).

Macrophages and dendritic cells play key roles in atherosclerosis progression and regression. One critical role is related to the process by which apoptotic cells (ACs) are cleared by phagocytic cells such as macrophages and dendritic cells, called efferocytosis (4, 5). Efferocytosis prevents secondary necrosis of ACs and subsequent inflammation and contributes to the production of pro-resolving mediators, which can feedback to further enhance efferocytosis (efferocytosis-resolution cycle) (4, 5). As atherosclerosis progresses, the efferocytosis-resolution cycle becomes defective, characterized by increased inflammation, enhanced necrosis, and thinning of the protective fibrous cap of lesions (4, 5). In humans, these changes lead to plaques that are “unstable”, meaning they increase the risk for plaque rupture or erosion, acute luminal thrombosis, and major adverse cardiovascular events, notably myocardial infarction, stroke, and sudden cardiac death (4, 5). On the other hand, the efferocytosis-resolution cycle is reawakened during atherosclerosis regression, a process in which marked lipid lowering leads to atherosclerotic plaque stabilization through necrotic core reduction and fibrous cap thickening and, in humans, lower the risk of coronary artery disease (6–8).

There is also substantial evidence indicating that T-cells from the adaptive immune system play important roles in atherosclerosis pathogenesis (9–12). Under homeostatic conditions and during an immune response, myeloid cells and T-cells communicate with each other (13–15), with macrophages and dendritic cells serving as a critical bridge between the innate and adaptive immune systems through antigen presentation, secreted mediators, and cell-cell contact (13–15). T-cells can also feedback through secreted factors and cell-cell contact to regulate myeloid cell functions (16, 17).

While the roles of myeloid cells in atherosclerosis are varied and complex, we focus here on the key issue of how efferocytosis by lesional macrophages and dendritic cells can affect both plaque progression and regression through crosstalk with T-cells. Crosstalk between efferocytic myeloid cells and T-cells can promote a pro-resolving milieu that dampens chronic inflammation and promotes plaque stabilization, while failure of this process can result in the exacerbation of atherosclerosis and attenuation of regression. In this review, we will explore three critical aspects of this interaction and how it may affect atherosclerosis progression and regression: 1) the role of antigen cross-presentation by myeloid cells of AC-derived antigens during efferocytosis and its effect on T-cells; 2) the role of secreted cytokines, such as transforming growth factor-β1 (TGF-β1) and interleukin-10 (IL-10), and metabolites, such as lactate and polyamines in the crosstalk between efferocytic myeloid cells and T-cells; and 3) the role of cell-cell contacts through membrane proteins, including the immune checkpoint proteins programmed death ligand 1 (PD-L1), programmed cell death protein 1 (PD-1), and cytotoxic T-lymphocyte associated protein 4 (CTLA-4) in regulating efferocyte-T-cell crosstalk.

## 2 The role of myeloid cell antigen presentation

Myeloid cells are adept at a process called antigen presentation. Proteins taken up by myeloid cells through efferocytosis and other endocytic pathways are degraded, and peptides (antigens) are loaded onto major histocompatibility complex (MHC) molecules to present to adaptive immune cells such as T-cells. The recognition of presented antigens by T-cells through the engagement of the T-cell receptor (TCR) can either trigger immune activation towards the antigen or induce tolerance toward self-antigens (18, 19). Disruption of tolerance mechanisms can result in aberrant presentation of self-antigens to auto-reactive T-cells and a lack of restraint on T-cell activation, leading to autoimmunity. Efferocytosis helps facilitate tolerance by preventing self-antigens derived from ACs from triggering an autoimmune response and chronic inflammation (20). Accordingly, an impairment in efferocytosis, which is observed in advanced atherosclerosis, can result in an accumulation of uncleared self-antigen thus inducing autoimmunity (4, 21). In late atherosclerosis, defective efferocytosis can be attributed in part to the loss of cell surface efferocytic receptors, *e.g.*, through the cleavage of the efferocytosis receptor, MER proto-oncogene tyrosine kinase (MerTK) (22). A direct example of autoimmunity triggered by impaired efferocytosis comes from a study showing that genetically deleting the efferocytic receptor CD300f in mice enhanced self-antigen presentation by dendritic cells, contributing to memory T-cell expansion and autoimmunity (23). Loss of tolerance due to impaired efferocytosis is likely relevant to atherosclerosis, which displays hallmarks of autoimmunity such as anti-apolipoprotein B autoantibodies and CD4^+^ T-cells (24, 25). This topic will be discussed in more detail in the following sections.

Antigen presentation is initiated when internalized proteins are degraded by lysosomes or by the proteasome and loaded onto MHC class I or II molecules and presented to a corresponding T-cell receptor (TCR). The proliferation and activation of T-cells can be initiated when TCRs on a CD4^+^ or CD8^+^ T-cell surpass the threshold binding affinity of a peptide presented on MHC class II or class I, respectively (26, 27). During efferocytosis, AC degradation generates self-antigens, but efferocytosing myeloid cells can restrain autoimmunity by suppressing the presentation of these antigens. The mechanism involves the secretion of pro-resolving mediators by efferocytes that (a) lower co-stimulatory signals needed for full activation of T-cells, *e.g.*, those that mediate T-cell CD28 interaction with myeloid CD80/86; and (b) promote the formation of self-antigen-specific forkhead box P3 (FoxP3)^+^ regulatory T-cells (T_reg_ cells), which suppress auto-reactive effector T-cells, dampen inflammation, and promote tissue repair (28–30). In atherosclerosis, FoxP3^+^ T_reg_ cells are athero-protective (31) and are critical to atherosclerosis regression (32). In dendritic cells, AC-binding and efferocytosis mediated by MerTK triggers the suppression of nuclear factor kappa B (NF_κ_B) signaling to prevent the full maturation of these cells (33). This process results in low expression of MHC, co-stimulatory molecules like CD80, and the pro-inflammatory cytokine tumor necrosis factor-alpha (TNF-α), all of which inhibit the presentation of AC-derived self-antigens and prevent the activation of auto-reactive T-cells (33). Pro-resolving mediators from efferocytes, such as TGF-β1 and IL-10, can induce T_reg_ cells, which feedback to suppress CD80/86 and stop effector T-cell activity (34).

In atherosclerosis, antigen-presentation by myeloid cells to T-cells contributes to chronic inflammation by inducing effector CD4^+^ T-cells and the release of pro-inflammatory cytokines, *e.g.*, IFN-γ and TNF-α, by activated T-cells (35). These cytokines in turn enhance the uptake of oxidized LDL (oxLDL) and minimally modified LDL by lesional macrophages, which promotes disease progression (36). Furthermore, pro-inflammatory cytokines such as IFN-γ and TNF-α are known to augment antigen presentation by myeloid cells, thus further activating pro-atherogenic effector T-cells (37, 38). Efferocytosis still occurs in inflammatory progressing atherosclerotic lesions, albeit reduced and with impaired induction of pro-resolution signaling. Thus, it is possible that in atherosclerosis progression, the suppression of antigen presentation and co-stimulatory molecules by efferocytes is impaired. For example, although antigenic peptides generated during efferocytosis can bypass being loaded onto MHC class II by the activity of Ras-related protein Rab-17 (Rab17) to avoid antigen presentation and activation of auto-reactive CD4^+^ T-cells (39), stimulation of toll-like receptors (TLRs) such as TLR4 can re-direct antigens to be loaded onto MHC molecules for antigen presentation (40–42). Thus, oxLDL, which accumulates in atherosclerotic plaques, can upregulate TLR4 expression and signal through TLR4 (43, 44), and other pro-inflammatory signals in atherosclerosis may enhance the presentation of self-peptide by efferocytosing myeloid cells to T-cells. Furthermore, chronic inflammation in atherosclerosis promotes the maturation of dendritic cells and expression of co-stimulatory molecules to enhance activation of self-reactive T-cells (45). Interestingly, MHC class II whole-body knockout in ApoE^-/-^ mice, designed to inhibit antigen presentation to CD4^+^ T-cells, worsened atherosclerosis despite reduced total CD4^+^ T-cells and circulating pro-inflammatory cytokines (46). This result may be related to the finding that antigen presentation by MHC class II of plaque antigens to T_reg_ cells is critical for immunosuppression (47), suggesting that a loss of T_reg_ cell activity is more consequential than reduced antigen presentation to effector CD4^+^ T-cells in atherosclerosis progression (46). Because these experiments were performed in whole-body MHC class II knockout mice and most cells can present antigen on MHC class II, it would be interesting to observe the effect on atherosclerosis when MHC class II is knocked out solely in myeloid antigen-presenting cells (46). In this context, mice with dendritic cell-specific knockout of the TLR signaling adaptor, myeloid differentiation primary response 88 (MyD88), attenuated dendritic cell maturation, antigen presentation to T_reg_ cells, T_reg_ cell maturation, and most importantly, worsened atherosclerosis (48). Thus, in this model of atherosclerosis, defective T_reg_ cell development trumped the benefits of reducing effector T-cells (48).

## 3 The role of secreted factors on myeloid-lymphocyte crosstalk

**Figure 1:**
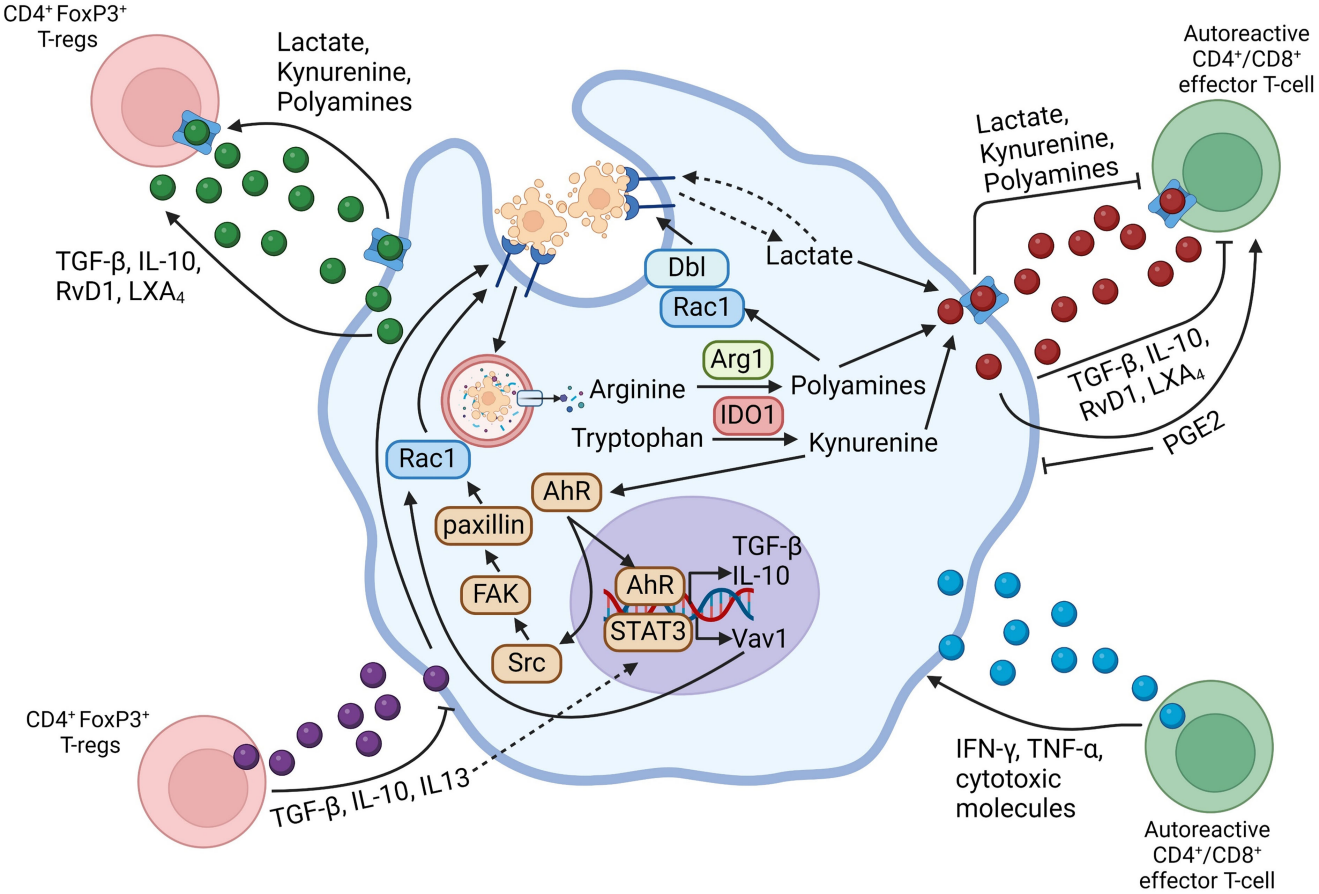
Schematic summarizing efferocytosing myeloid cell-T-lymphocyte crosstalk through secreted mediators. Arrows going from the myeloid cell-secreted molecule to the T-lymphocyte indicate that the secreted mediator activates the functions of the indicated T-lymphocyte subset, whereas flat-ended lines indicate an inhibitory function of the secreted molecule towards the indicated T-lymphocyte subset. Arrows going from the T-lymphocyte-secreted molecule towards the myeloid cell indicate a pro-inflammatory function on the myeloid cell, whereas flat-ended lines indicate a pro-resolving function of the secreted molecule on the myeloid cell. Figure generated with BioRender.com.

As mentioned above, both efferocytes and activated T-lymphocytes can produce a variety of cytokines and lipid-derived specialized pro-resolving mediators (SPMs). In addition, efferocytes release metabolites derived from the degradation of ACs and from shifts in cellular metabolism, and these molecules could have immunomodulatory effects on both the efferocyte and T-cells. The role of secreted molecules in efferocytosing myeloid cell-lymphocyte crosstalk is summarized in **Figure 1**.

### 3.1 Cytokines

Efferocytosis of ACs promotes the secretion of pro-resolving cytokines such as TGF-β1 and IL-10. TGF-β1 is a key signal in driving the polarization of CD4^+^ CD25^+^ T-cells to FoxP3^+^ T_reg_ cells by inducing FoxP3 expression and stabilization (49). In the thymus, negative selection and subsequent thymocyte apoptosis drives enhanced thymic FoxP3^+^ T_reg_ cell differentiation due to TGF-β1 production resulting from efferocytosis by thymic myeloid cells (50). IL-10 can also maintain FoxP3 expression and promote the immunosuppressive activity of FoxP3^+^ T_reg_ cells (51). TGF-β1 and IL-10 also play a role in the suppression of the pro-inflammatory T_H_1 effector response (52, 53). TGF-β1 can inhibit the expression of T-bet and IRF1, which are lineage-specific transcription factors for pro-inflammatory T_H_1 cells, through the activity of the protein tyrosine phosphatase Src homology region 2 domain-containing phosphatase-1 (Shp-1) (54), and IL-10 plays a critical role in restraining T_H_1 cell activity (53). Alloreactive/autoreactive CD4^+^ T-cells pre-treated with TGF-β1 and IL-10 are converted into T_reg_ cells, preventing an aberrant T-cell response, while increased T_reg_ cell numbers help maintain immunosuppression and generate a pro-resolving environment (55, 56). Thus attenuated TGF-β1 and IL-10 accumulation in atherosclerotic lesions due to impaired efferocytosis may result in a lower threshold to T-cell activation, thus resulting in pro-atherosclerotic autoimmunity and excessive T-cell activity. However, the actions of TGF-β1 are complex. On the one hand, TGF-β1 can enhance the activation and proliferation of T_H_1 cells, but TGF-β1 can also enhance IL-10 production by T_H_1 cells to limit this effect (57). As another example, TGF-β1 can suppress naïve CD8^+^ cytotoxic T-cells (58, 59), though TGF-β1 supports the activation and survival of memory or effector CD8^+^ T-cells (58). This dichotomy suggests that TGF-β1 prevents aberrant CD8^+^ T-cell activation, but will support a CD8^+^ response in clones that under normal conditions are appropriately responsive to a threat. In later stages of atherosclerosis, CD8^+^ cytotoxic T-cells that show specificity to a limited set of plaque-associated antigens remain and constitute a large proportion of plaque immune cells, likely due to impaired suppression of allo/auto-reactive CD8^+^ T-cells (60). CD8^+^ T-cells then contribute to necrotic core growth and plaque destabilization by the cytotoxic killing of cells in the plaque (61) and by secreting pro-inflammatory cytokines such as IFN-γ (62).

Cytokines secreted by T-cells can also feedback to enhance efferocytosis and generate a pro-resolving phenotype in myeloid cells, such as FoxP3^+^ T_reg_ cells secreting pro-resolving TGF-β1 and IL-10 (63). Both TGF-β1 and IL-10 signal to macrophages to enhance efferocytosis and polarize macrophages towards a pro-resolving phenotype (64, 65). Thus, this could be a mechanism by which efferocytosing myeloid cells drive a positive feedback loop by activating FoxP3^+^ T_reg_ cells with TGF-β1 and IL-10, which further enhances TGF-β1 and IL-10 levels through T_reg_ cells. In addition to TGF-β1 and IL-10, FoxP3^+^ T_reg_ cells could also promote efferocytosis by IL-13 (17). IL-13 signaling in macrophages stimulates IL-10 production by macrophages, which then signals in an autocrine/paracrine manner leading to signal transducer and activator of transcription 3 (STAT3)-mediated upregulation of the small Rho GTPase Vav1 to promote actin mobilization through Ras-related C3 botulinum toxin substrate 1 (Rac1) and enhanced efferocytosis (17).

### 3.2 Metabolites

Efferocytosing macrophages rewire their metabolism by enhancing glycolysis and fatty acid oxidation, and increase the expression and activity of enzymes that process metabolic cargo like amino acids released from the degradation of ACs. Metabolites released by macrophages can modulate the survival, proliferation, differentiation, and activity of T-cells.

#### 3.2.1 Lactate

Recent studies have shown that efferocytosis causes a transient increase in aerobic glycolysis and lactate production in macrophages, which is distinct from the prolonged nature of these responses in inflammatory macrophages (66–68). Efferocytosis promotes glycolysis through phosphorylation and activation of the kinase PFKFB2 and increased glucose uptake by stabilizing GLUT1 (67). Efferocytosis-induced lactate can be secreted into the extracellular space by the lactate transporter SLC16A1 to signal to neighboring cells (66–68). Secreted lactate regulates the differentiation, proliferation, and activity of multiple T-cell subsets (69). FoxP3^+^ T_reg_ cells efficiently utilise lactate rather than glucose as a fuel source by shuttling lactate into the TCA cycle, which sustains the immunosuppressive functions of T_reg_ cells and promotes their expansion (70). Lactate was found to also promote the expression of FoxP3 in naïve T-cells resulting in their polarization to FoxP3^+^ T_reg_ cells (71). Pro-inflammatory effector T-cells, however, are inhibited from proliferation by lactate due to a reduction in the NAD^+^/NADH ratio, which promotes reductive stress (72). Under conditions of chronic inflammation, such as in a mouse model of rheumatoid arthritis where lactate accumulates in the microenvironment, CD4^+^ effector T-cells metabolically adapt by upregulating the lactate transporter, SLC5A12 (73). This process exacerbates inflammation by (a) increasing lactate import, which promotes the expression of the transcriptional regulator of the T_H_17 lineage, RORγT; (b) boosting the production of the pro-inflammatory cytokine IL-17 though PKM2 dimer-STAT3 signaling and enhanced fatty acid synthesis, which further enhances T_H_17 polarization (73). These findings also indicate that the duration of glycolytic lactate production and concentration of lactate can influence T-cell polarization and activity. A more transient increase in glycolysis by efferocytic macrophages may support FoxP3^+^ T_reg_ cells and suppress effector T-cells to dampen inflammation, while chronic inflammation may result in metabolic adaptations by effector T-cells that lead to the induction of pro-inflammatory signals.

In murine atherosclerosis, myeloid-specific knockout of GLUT1 in Western diet-fed *Ldlr^-/-^* mice resulted in expanded necrotic cores and accumulation of ACs due in part from reductions in efferocytosis resulting from impaired glycolysis and lactate production (66). Moreover, the circulating lactate concentration in humans correlates with atherosclerosis severity (74). Thus, it will be interesting to determine in the future if perturbations in glycolytic metabolism and lactate production affect the activity of effector T-cells and the differentiation of T_reg_ cells in atherosclerotic plaques.

#### 3.2.2 Polyamines

Polyamines are a class of metabolites derived from the enzymatic conversion of the amino acid L-arginine. L-arginine is converted by the enzymes Arginase 1/2 to L-ornithine, which is subsequently converted to either L-proline to support collagen synthesis or other polyamine species including putrescine, spermidine, and spermine, through ornithine decarboxylase 1 (ODC1) and other downstream enzymes (75). Efferocytosing macrophages increase intracellular L-arginine, which is derived from degraded ACs, and convert it to polyamines such as ornithine and putrescine (76). Putrescine generated after the uptake of a first AC was found to enhance continuing efferocytosis by increasing the expression of the GTPase Dbl to activate Rac1 and subsequent actin remodeling for second AC internalization (76). Other studies have also identified that efferocytosing macrophages can enhance the import of spermidine and spermine to suppress pro-inflammatory cytokine production (e.g. IL-1β and IL-6) in LPS-treated macrophages (77).

Arginine and polyamines can be exported by amino acid and diamine transporters such as SLC3A2 (78). T-cells have the capacity to metabolize arginine to polyamines and import polyamines to modulate their behavior. *In vitro* supplementation of T-cells with spermidine enhanced FoxP3 expression and enhanced polarization of T-cells to T_reg_ cells (79). Administration of L-arginine or spermidine to mice increased T_reg_ cell content *in vivo* (79). Spermidine and its conversion to hypusine was also shown to be necessary for maintaining T-cell lineage fidelity through hypusination of the translation elongation factor eIF5A, which is necessary for chromatin remodeling to ensure proper T-cell lineage commitment (80). ODC1 KO in CD4^+^ T-cells resulted in dysregulated expression of lineage-specific transcription factors and cytokines in several T-helper cell subsets, and adoptive transfer of ODC1 KO CD4^+^ T-cells into *Rag1* ^-/-^ mice exacerbated inflammation and disease progression in a colitis model (80). These data suggest that polyamines regulate CD4^+^ T-cell pro-inflammatory signaling in inflammatory diseases. By blocking both polyamine biosynthesis through ODC1 KO and polyamine uptake through the polyamine mimetic AMXT 1501 to fully deplete intracellular polyamines, T-cell proliferation and differentiation toward T_H_1 and T_H_17 was inhibited, while differentiation towards T_reg_ cells was increased (81). In a mouse experimental autoimmune encephalopathy (EAE) model of autoimmune disease, a combination of ODC1 KO donor T-cells and daily injections of AMXT 1501 almost completely suppressed disease progression (81). ODC1 expression is distinct between different T-cell subsets, suggesting that variations in polyamine synthesis guide and maintain lineage fidelity rather than triggering CD4^+^ T-cell polarization towards a particular lineage (80). In CD8^+^ T-cells, polyamines can support immunosuppression. Spermidine was shown to prevent TCR clustering on the cell membrane by cholesterol depletion to inhibit CD8^+^ T-cell activation (82). Furthermore, spermidine and hypusination of eIF5A in effector CD8^+^ T-cells prevented the formation of tissue-resident memory CD8^+^ T-cells and reduced the production of the pro-inflammatory cytokines TNF-α and IFN-γ (83).

In atherosclerosis, macrophage-specific deletion of arginase-1 in *Ldlr^-/-^* mice did not affect atherosclerosis progression, but it significantly attenuated atherosclerosis regression as assessed by an increase in plaque area and thinning of the fibrous cap (76). Given that arginase-1 is upregulated in pro-resolving/efferocytic macrophages, this may explain the importance of arginase-1 and polyamine production in regressing lesions, which are characterized by enhanced efferocytosis. Overexpression of arginase-1 in macrophages resulted in the suppressed production of pro-inflammatory cytokines such as TNF-α and IL-6 *in vitro* and *in vivo* in the atherosclerotic plaque (84). Both IL-6 and TNF-α can contribute to the proliferation of pro-atherogenic T-cell subsets including CD4^+^ T_H_1 and T_H_17 cells (85, 86), suggesting that macrophage arginase-1, perhaps through ornithine-derived polyamines, may contribute to ameliorating atherosclerosis progression by suppressing these T-cell subsets from differentiating and expanding. In atherosclerosis progression, direct supplementation of the polyamine putrescine to the drinking water was able to reduce lesion area and necrotic core area while increasing fibrous cap thickness (76). This was found to be due in part to enhanced continual efferocytosis from putrescine-mediated upregulation of the small GTPase Dbl (76). However, an additional mechanism could be that putrescine and other polyamines produced from AC-derived arginine can also play a role in increasing FoxP3^+^ T_reg_ cell activity and reducing effector T-cells to attenuate atherosclerosis progression and enhance atherosclerosis regression.

#### 3.2.3 Tryptophan-derived metabolites

The amino acid tryptophan can be converted into several downstream bioactive metabolites by myeloid cells. Tryptophan is converted by indoleamine 2,3-dioxygenase 1 (IDO1) to kynurenine, which is the central metabolite to produce other tryptophan-derived molecules. Efferocytosing macrophages can utilise tryptophan derived from the degradation of ACs to generate downstream metabolites. Kynurenine is a key activator of the transcription factor, aryl hydrocarbon receptor (AhR), leading to its release from chaperone proteins that keep AhR in the cytoplasm and its nuclear translocation to bind to gene promoters at aryl hydrocarbon responsive elements (AHREs) (87). AhR activity is known to enhance pro-resolution signaling in macrophages (88). In efferocytosing macrophages, activation of AhR increased the production of pro-resolving mediators such as TGF-β1 and IL-10 and suppressed the production of pro-inflammatory cytokines such as TNF-α and IL-1β (88). In a mouse model of lupus, AhR inhibition with the antagonist CH223191 was found to exacerbate autoimmunity (88).

Metabolites such as kynurenine can be exported and imported through transporters such as SLC7A5 (LAT-1), SLC7A11, or SLC36A4 (PAT-4) by myeloid cells and T-cells (89–91). Kynurenine and related metabolites play an important role in the differentiation and activity of T-cells. Macrophage-derived tryptophan metabolites can suppress the proliferation of activated T-cells (92), and the uptake of extracellular IDO1-dependent tryptophan metabolites by CD8^+^ T-cells was found to be necessary for their proliferation following activation (90). In the regulation of T-cell differentiation, kynurenine can be imported to activate AhR and induce a pro-resolving phenotype by enhancing the T_reg_ cell-specific transcription factor, FoxP3 (93, 94). However, some evidence suggests that AhR can promote both T_reg_ cell and pro-inflammatory T_H_17 differentiation depending on the context (95).

Tryptophan-derived metabolites play a role in the pathogenesis of atherosclerosis. In mice, IDO1 activity in dendritic cells and T_reg_ cell activity are mutually supportive in reducing atherosclerosis (96). This suggests a role of secreted tryptophan-derived metabolites from dendritic cells to enhance T_reg_ cell pro-resolving functions and a role of T_reg_ cells in increasing the production of tryptophan-derived metabolites (96). In human atherosclerosis, a decrease in the expression of enzymes within the plaque that are necessary to generate AhR ligands is correlated with an unstable plaque and exacerbation of atherosclerosis (97).

### 3.3 Lipid mediators

Efferocytosis promotes the production and secretion of a group of lipid pro-resolving mediators called SPMs, which are synthesized from arachidonic acid (AA) and docosahexaenoic acid (DHA). Pro-inflammatory lipid mediators can also be generated by the same precursors depending on the activity of downstream enzymes, and the ratio of pro-resolving:inflammatory mediators is a determinant of whether inflammation resolution and tissue repair versus inflammatory tissue damage occurs. Prominent SPMs produced by efferocytosing myeloid cells include resolvin D1 (RvD1), lipoxin A4 (LXA_4_), and prostaglandin E2 (PGE2) (64, 98, 99). Although PGE2 signaling has been shown to have pro-resolving effects in the context of efferocytosis, it has also been shown to have a pro-inflammatory effect in other contexts (64, 100, 101). RvD1, LXA_4_, and PGE2 have been shown to act on other myeloid cells in an autocrine-paracrine manner to promote a pro-resolving and pro-efferocytic phenotype (64, 100, 102–105). Efferocytosis-induced PGE2 can also act on dendritic cells to inhibit their maturation and antigen presentation capacity, which prevents the activation and proliferation of T-cells in autoimmunity (106). SPMs are also able to mediate pro-resolving signaling in T-cells. RvD1 and LXA_4_ signaling can enhance T-cell differentiation to T_reg_ cells while suppressing the differentiation to pro-inflammatory T_H_1 and T_H_17 cells (107, 108). However, PGE2 signaling through the EP2 and EP4 receptors resulted in the inhibition of TGF-β-mediated FoxP3 expression in naïve T-cells, which blocked T_reg_ cell differentiation, and enhanced pro-inflammatory T_H_1 and T_H_17 differentiation (109–111). Thus, PGE2 seems to have antagonistic effects suppressing pro-inflammatory polarization in macrophages while supporting a pro-inflammatory phenotype in T-cells.

In atherosclerosis, SPM levels are known to decrease in both mice and humans (112, 113). Specifically, a decrease in the ratio of SPMs to pro-inflammatory lipid mediators is associated with plaque instability and plaque progression (112, 113). Increasing this ratio by exogenous administration of RvD1 was found to enhance features of plaque stability in mice (112). As mentioned above, the effects of PGE2 are complex, and this applies to atherosclerosis (114, 115), i.e., the relative balance or its ability to promote a pro-resolving phenotype in myeloid cells and to polarize T cells toward a T_H_1/T_H_17 cell versus T_reg_ cell phenotypes. In addition, PGE2 may have effects on other cell types within the plaque that influence atherosclerosis progression or regression.

## 4 The role of cell-cell contacts between efferocytes and T-cells

**Figure 2:**
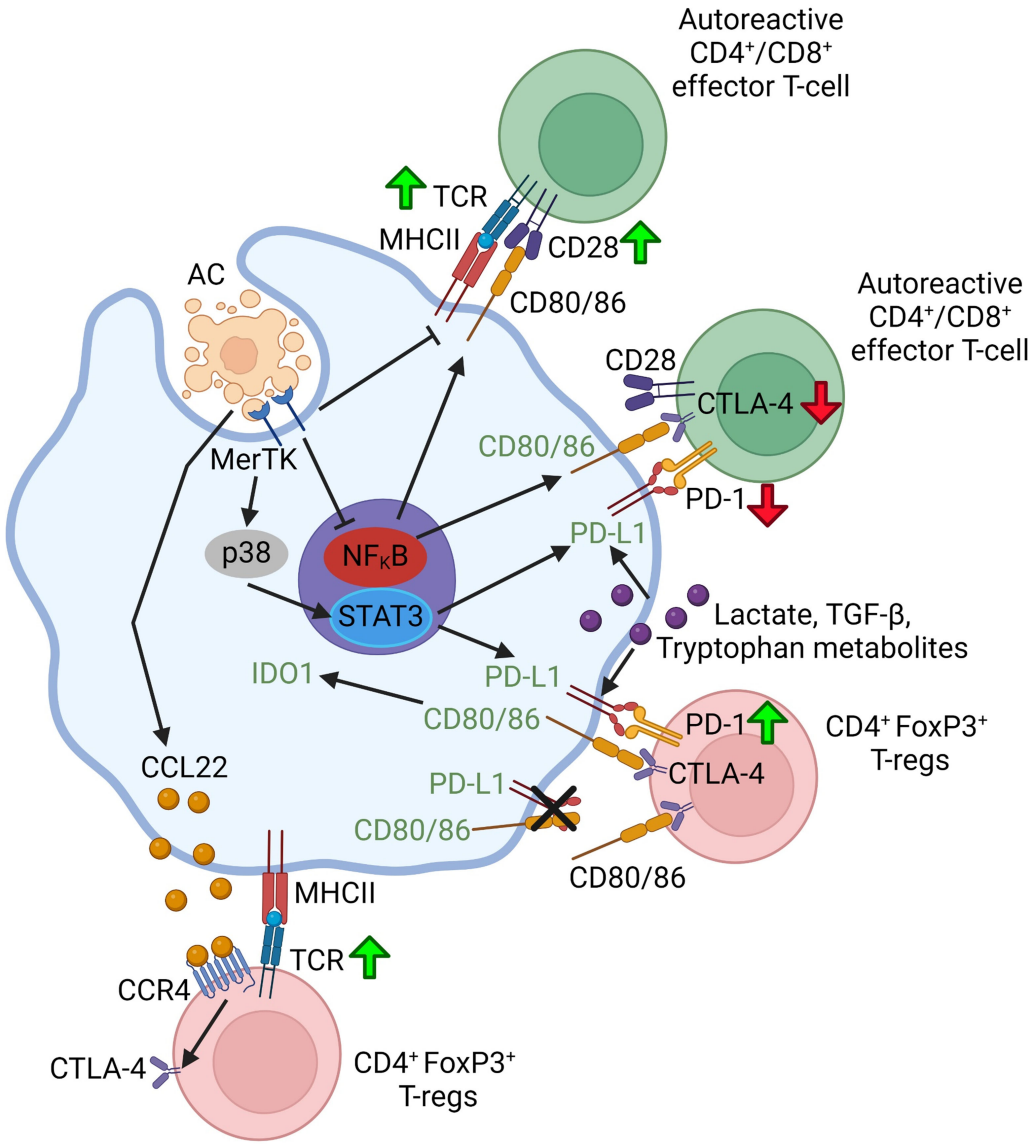
Schematic summarizing efferocytosing myeloid cell-T-lymphocyte crosstalk through cell-cell contact. Green up-arrows indicate that a particular molecular interaction activates the function of the indicated T-lymphocyte subset or promotes the differentiation of naïve T-lymphocytes to that subset. Red down-arrows in T-cells indicate an inhibition of function or differentiation. Green font in the myeloid cell indicate that a particular molecular interaction promotes a pro-resolving myeloid cell phenotype or function. Figure generated with BioRender.com.

Macrophages can affect T cells through cell-cell contact involving interaction between the checkpoint proteins on the two cell types, notably PD-L1 and PD-1, and cytotoxic T-lymphocyte associated protein 4 (CTLA-4). The role of cell-cell contacts in efferocytosing myeloid cell-lymphocyte crosstalk is summarized in **Figure 2**. PD-L1 is expressed more ubiquitously including on macrophages and dendritic cells, whereas PD-1 is primarily expressed on T-cells (116). Interaction between PD-L1 and PD-1 can suppress the activation of effector CD4^+^ and CD8^+^ T-cells while promoting the differentiation of naïve CD4^+^ T-cells to FoxP3^+^ T_reg_ cells and sustaining T_reg_ cell activity, in part by upregulating and sustaining FoxP3 expression (16, 117). Through MerTK-p38-STAT3 signaling, macrophages upregulate PD-L1 upon binding ACs (118). Moreover, PD-1 expression on FoxP3^+^ T_reg_ cells and cytotoxic CD8^+^ T-cells, and PD-L1 expression on macrophages can be upregulated by secreted factors from efferocytosing macrophages such as lactate (119), TGF-β1 (120), and tryptophan-derived metabolites (91).

There is also evidence that efferocytosis by myeloid cells may also induce the expression of another immune checkpoint protein, CTLA-4, on T_reg_ cells. CTLA-4 is a cell surface protein that is expressed principally on T-cells, but it can also be expressed by other immune and non-immune cell types such as myeloid cells and tumor cells. CTLA-4 expression is induced and sustained on T_reg_ cells and memory T-cells following the interaction of the T-cell receptor (TCR) and CD28 on activated T cells with the MHC-peptide complex and CD80/86 on antigen-presenting cells, such as macrophages and dendritic cells (121). CTLA-4 suppresses signaling downstream of the TCR to shut off T-cell proliferation and the T-cell response in effector T-cells (122, 123). On T_reg_ cells, CTLA-4 expression is critical to their tolerogenic and immunosuppressive function in several ways (124–126). Firstly, CTLA-4 can compete with CD28 for binding to CD80/86 on antigen-presenting cells to reduce T-cell activity (127). Secondly, CTLA-4 can mediate “transendocytosis” of CD80/CD86, which is the process by which T_reg_ cells engulf and remove CD80/86 from the antigen-presenting cell (125, 128, 129). This degradation can prevent CD28 co-stimulation as well as disrupt cis-CD80-PD-L1 dimer interactions on the antigen presenting cell (125, 128, 129). CD80 dimerization with PD-L1 prevents PD-1 on other cell types such as T-cells from interacting with PD-L1 to induce tolerogenic signaling, and thus disruption of the dimers allows for restoration of PD-L1-PD-1 signaling (125). Thirdly, administration of CTLA-4-Ig, which mimics CTLA-4 binding and its interaction with macrophage CD80/86, can suppress macrophage pro-inflammatory cytokine production and promote phenotypic switching to a pro-resolving phenotype (130, 131). Although not directly shown using T_reg_ cells, this may be a potential mechanism for T_reg_ cell-mediated inflammation resolution. Finally, T_reg_ cell CTLA-4 can induce dendritic cells to express IDO1 and increase tryptophan catabolism in a CD80/86-dependent manner (132). This increase in IDO1 (a) enhances the secretion of kynurenine and other tryptophan-derived metabolites by dendritic cells to modulate efferocytosing myeloid cells and T-cells as described previously; and (b) suppresses antigen presentation by dendritic cells (132). In the context of efferocytosis, CD169^+^ splenic marginal zone macrophages upregulated the secretion of CCL22, the chemotactic ligand for CCR4, following the uptake of apoptotic thymocytes *in vitro* (133). *In vivo*, injection of ACs into mice upregulated CCL22 secretion by CD169^+^ splenic macrophages, and T_reg_ cells were found to migrate into the spleen and increase CTLA-4 expression in a CCR4-dependent manner (133). These findings suggest that CCL22-CCR4 signaling or signaling by another secreted factor produced by efferocytosing macrophages, can increase CTLA-4 expression on T_reg_ cells. Moreover, CTLA-4 can be upregulated in T cells, including T_reg_ cells, by pro-resolving lactate and TGF-β (120, 134), but more work is needed to understand how CTLA-4 expression is affected by efferocytosing myeloid cells and how T-cell CTLA-4 signals back to efferocytosing myeloid cells.

In murine models of atherosclerosis progression, whole-body knockouts of either PD-L1 or PD-1 resulted in an exacerbation of atherosclerosis progression as measured by plaque area (135, 136). This corresponded with an increase in the number of macrophages, CD4^+^ T-cells, and CD8^+^ T-cells (135, 136). *In vitro*, myeloid cells from PD-L1 KO mice could more readily activate T-cells following antigen presentation, and T-cells from PD-1 KO mice were more inflammatory and cytotoxic in the case of CD8^+^ T-cells (135, 136). Interestingly, hypercholesterolemia increased PD-L1 expression on myeloid cells and increased PD-1 expression on T-cells, suggesting a possible compensatory response in which PD-L1-PD-1 interaction restrains the inflammatory response during atherosclerosis progression (135, 136). In this context, cancer patients who receive blocking antibodies targeting PD-L1 or PD-1 have an aggravation of atherosclerotic cardiovascular disease (137).

CTLA-4 was also found to have an anti-atherogenic effect in mice and humans (138–141). CTLA-4 blocking antibodies, similar to PD-L1/PD-1 blocking antibodies, are used to treat cancer, which is associated with an increased risk of coronary artery disease (138, 141). In Western diet-fed *Ldlr^-/-^* mice, CTLA-4 blockade increased effector CD4^+^ and CD8^+^ T-cells in the blood, spleen, and plaque, increased plaque size, and decreased features of plaque stability based on increased necrotic core size and thinner fibrous caps (139). Conversely, transgenic T-cell overexpression of CTLA-4 resulted in an attenuation of atherosclerotic plaque size in the aortic root, with a reduced accumulation of macrophages and effector T-cells in the plaque (140). Furthermore, dendritic cell maturation was inhibited as determined by reduced CD80/86 expression, although this could at least in part be a result of enhanced transendocytosis of CD80/86 by T_reg_ cells (140).

## 5 Conclusion

Both myeloid cells and T-cells play important roles in affecting the plaque microenvironment in atherosclerosis progression and regression by modulating inflammation and other immune processes. Significant crosstalk occurs between myeloid cells and T-cells to influence the phenotype and behavior of both groups of immune cells. Efferocytosis by myeloid cells is a critical component of inflammation resolution and tissue repair, affecting the metabolism, secretome, and gene expression of macrophages and dendritic cells. These changes in efferocytes can lead to altered interactions between myeloid cells and T-cells and phenotypic alterations in T-cells. T-cells can also feedback to regulate efferocytes, thus contributing to the perpetuation or disruption of the efferocytosis-resolution cycle. In atherosclerosis, impaired efferocytosis exacerbates inflammation and increased plaque destabilization, whereas improved efferocytosis in atherosclerosis regression reversed these changes. Although the pro-tolerogenic function of myeloid cell efferocytosis and its associated downstream signals, *e.g.*, secreted lactate or kynurenine, are beneficial in the context of chronic inflammation and atherosclerosis progression, this process can also contribute to immunosuppression in cancer, leading to cancer cell evasion of the immune system (142–145). Thus, conceptualizing methods to target the crosstalk between efferocytosing myeloid cells and T-cells for the treatment of atherosclerosis must also consider that excessive immunosuppression may result in impaired anti-tumor immunity. Thus, a deeper understanding of efferocyte-T-cell crosstalk is crucial to better understand how to achieve a balance between pro-inflammatory signaling and pro-resolution signaling. Future analysis of publicly available datasets or using tools such as CellChat to analyse cell-cell interactions could be used to identify candidate signaling pathways to target to better achieve the balance (146).

In this review, we introduce mechanisms by which efferocyte-T-cell crosstalk through antigen presentation, secreted factors, and cell-cell contact by ligand-receptor interactions could contribute to the benefits of efferocytosis on inflammation resolution and plaque stability. Much remains to be discovered about this interplay, and further work in this area has the potential to provide new insights into the pathogenesis of atherosclerosis and to suggest novel therapeutic targets.

## Author contributions

DN: Conceptualization, Investigation, Writing – original draft, Writing – review & editing. SS: Investigation, Writing – review & editing. IT: Supervision, Writing – review & editing.
